# Discrimination of emotional states from scalp- and intracranial EEG using multiscale Rényi entropy

**DOI:** 10.1371/journal.pone.0186916

**Published:** 2017-11-03

**Authors:** Yelena Tonoyan, Theerasak Chanwimalueang, Danilo P. Mandic, Marc M. Van Hulle

**Affiliations:** 1 Research Group Neurophysiology, Laboratory for Neuro- and Psychophysiology, Leuven, Belgium; 2 Communication and Signal Processing Research Group, Department of Electrical and Electronic Engineering, Imperial College London, London, United Kingdom; Radboud Universiteit, NETHERLANDS

## Abstract

A data-adaptive, multiscale version of Rényi’s quadratic entropy (RQE) is introduced for emotional state discrimination from EEG recordings. The algorithm is applied to scalp EEG recordings of 30 participants watching 4 emotionally-charged video clips taken from a validated public database. Krippendorff’s inter-rater statistic reveals that multiscale RQE of the mid-frontal scalp electrodes best discriminates between five emotional states. Multiscale RQE is also applied to joint scalp EEG, amygdala- and occipital pole intracranial recordings of an implanted patient watching a neutral and an emotionally charged video clip. Unlike for the neutral video clip, the RQEs of the mid-frontal scalp electrodes and the amygdala-implanted electrodes are observed to coincide in the time range where the crux of the emotionally-charged video clip is revealed. In addition, also during this time range, phase synchrony between the amygdala and mid-frontal recordings is maximal, as well as our 30 participants’ inter-rater agreement on the same video clip. A source reconstruction exercise using intracranial recordings supports our assertion that amygdala could contribute to mid-frontal scalp EEG. On the contrary, no such contribution was observed for the occipital pole’s intracranial recordings. Our results suggest that emotional states discriminated from mid-frontal scalp EEG are likely to be mirrored by differences in amygdala activations in particular when recorded in response to emotionally-charged scenes.

## 1 Introduction

The identification and discrimination of emotional states from EEG is considered notoriously challenging, mainly due to the difficulty to gauge electromagnetic activity elicited by cortical structures involved in processing emotional information [1], yet potentially useful for a broad range of important applications such as diagnosing and treating patients with dysfunctional processing of emotional information [2], emotion-sensitive interactive games, affective interfaces, and emotion-sensitive tutoring systems [3–5]. This article sets out to address these challenges, but rather than evaluating the power in the standard EEG frequency bands, as is traditionally done [6–13], or amplitudes and latencies of event-related potentials (ERPs) in response to emotion-evoking stimuli [14–19], we conduct the analysis within the realm of complexity science [20].

Several entropy-based metrics of signal complexity have already been proposed for discriminating emotional states. Aftanas and co-workers [21] showed that, when viewing images evoking negative and positive emotions, higher values of EEG correlation dimension complexity are observed compared to viewing neutral images. Hosseini and Naghibi-Sistani [22] applied two entropic metrics (approximate and wavelet entropy) to discriminate between two emotional states (calm-neutral and negative-excited) in response to viewing sequences of emotion inducing pictures and achieved 73.25% classification accuracy. Jie et al. [23] applied sample entropy to EEG data obtained from two binary emotion recognition tasks (positive vs. negative emotion both with high arousal, and music clips with different arousal levels) and achieved 80.43% and 79.11% classification performance.

A promising development in signal complexity analysis is Rényi entropy (RE). Originally introduced as a generalization of Shannon entropy [24], RE has enjoyed several successful EEG-based clinical applications [13,25]. However, RE has been less utilised for EEG-based mental and affective state detection. Sourina and co-workers [26] used an RE-based variant (Hausdorff dimension) to calculate the fractal dimension of EEG for real time emotion quantification and classification, and Bajaj and Pachori [27] used RE together with other EEG complexity measures for emotion detection.

However, one should also be aware that, at least according to some authors [28], neither strictly periodic nor completely random signals should be regarded complex but rather signals that possess long range correlations across multiple temporal scales. As the cited studies assess entropy on a single scale, and since the used entropy metrics become maximal for random signals, their outcome could be confounding randomness with complexity. To avoid this confusion, Costa and co-workers [29] accounted for the interdependence between entropy and scale and proposed to calculate entropy, in their case sample entropy, on different temporal scales (whence, multiscale sample entropy, MSE), using the so-called coarse graining approach (averaging the signal over non-overlapping windows of increasing length). However, as the latter factually corresponds to a smoothing operation, predominantly low-frequency signal components were captured. To overcome this, empirical mode decomposition (EMD) [30] and its multivariate extension (MEMD) [31] have been suggested: a fully data-driven, time-frequency technique that decomposes a signal into a finite set of amplitude/frequency modulated components, called intrinsic mode functions (IMFs). A further improvement is multivariate MSE (MMSE), to account for both within and cross-channel dependencies, further combined with MEMD into MEMD-enhanced MMSE [32]. When applying EMD to EEG recordings, entropy can be estimated in each IMF individually. Sharma et al. [33] recently applied this technique to several entropic complexity measures, including RE, to predict focal epileptic seizures from EEG. Also recently, Tonoyan and co-workers [34] used MEMD in combination with MSE to discriminate 5 emotional states from mid-frontal EEG recordings when viewing emotionally charged video clips compiled by Schaefer and co-workers [35]. Albeit the results were encouraging, MSE-based entropy estimation turned out not only to be sensitive to the choice of window length but also computationally intensive.

Our aim for this study is to curb these drawbacks by revisiting MEMD-based EEG signal complexity in response to emotionally charged video clips, but now based on Rényi’s quadratic entropy (RQE) applied to whole scalp recordings of 30 subjects. We use Krippendorff’s inter-rater statistic [36] to identify the scalp electrodes that best discriminate between emotional states across subjects. We also recorded intracranial EEG (iEEG) of the amygdala and the occipital cortex jointly with scalp EEG of a patient viewing emotional and neutral video clips. The amygdala is considered to be strongly involved in emotional processing [37–48]. Motivated by Makeig and co-workers [49], who considered similar signal processing techniques for analyzing Human Intracranial Electrophysiology (HIE) and scalp EEG, we apply the same MEMD-based RQE method to the intracranial recordings and verify whether we can discriminate between the neutral and the emotional movies in a way similar to the jointly recorded scalp EEG.

## 2. Materials and methods

### 2.1 Materials

#### 2.1.1 Video clips

We considered emotionally-charged video clips of the public database developed by Schaefer and co-workers [35] (http://nemo.psp.ucl.ac.be/FilmStim/) (Table 1). The spoken language of these video clips was French or dubbed into French. Each video clip in the database is labeled in terms of emotional category (fear, anger, sadness, disgust, amusement, tenderness, neutral, further called “standard label”), which Schaefer and co-workers obtained by asking their participants to report what they personally felt in reaction to the video clips, not what they believed people would generally feel. The average duration of all video clips was approximately 3 minutes.

**Table 1:** Names, labels and affect scores of the video clips used in our main and control experiments. Name of videos between quotes and scene numbers between round brackets, standard labels between square brackets, positive or negative emotional affect scores (mean scores between round brackets), and our participants’ self-labels (underlined followed by the number of respondents between brackets). Video clips were taken from http://nemo.psp.ucl.ac.be/FilmStim/, standard labels and affect scores from Table 1 in [35] or provided by Alexandre Schaefer (personal communication).

Videos		[Standard label] self-labels		Affect scores	
Main	Control	Main	Control	Main	Control
‘Sleepers’	‘Schindler’s List (3)’	[Anger] Anger(14)/Disgust (16)	[Anger] Anger (6)	Negative (2.46)	Negative (2.14)
‘Life is beautiful’ (4)	‘The eight day’	[Tenderness] Tenderness (30)	[Tenderness] Tenderness (6)	Positive (2.49)	Positive (2.07)
‘City of angels’	‘Life is beautiful’ (1)	[Sadness] Sadness (30)	[Sadness] Sadness (6)	Negative (1.51)	Negative (2.01)
‘La cité de la peur’	‘The visitors’	[Amusement] Amusement (30)	[Amusement] Amusement (6)	Positive (2.31)	Positive (2.25)

We performed two experiments, further called main and control. For our main experiment, we selected two sets of 4 video clips (out of 70 video clips), all with top ten scores in their respective emotional categories. For the first set, 3 out of 4 videos (disgust, amusement, tenderness) had, in addition, also top ten positive or negative affect scores (cf., Table 1); the 4^th^ video (sadness) did not have a top ten affect score. It was selected to verify the statement of Aftanas and co-workers [21] that higher complexity values can be observed for affective stimuli compared to more neutral ones. As these videos have different emotional labels and affect scores, we want to see whether this translates into a difference in EEG complexity values. For the second study, 4 video clips were chosen but not from the top ten affect score list: as these videos shared lower affect scores, we used them in our experiment as controls. All videos were presented in random order to our participants.

For the implanted patient, as the implant serves a medical purpose, the time window that we could dispose of to perform experiments was restricted. Hence, we used only two video clips: fragment 3 of ‘Schindler’s list’ as an emotional video (standard labeled as ‘anger’) and the weather forecast as an example of a more neutral video. The data recorded in this patient was used as a case study for assessing the differential activation of the amygdala in response to the two video clips and the putative relation between amygdala activation and scalp EEG.

After watching each video clip, all participants (including the implanted patient) were asked to categorize the evoked emotion (after providing them with the same list as used in Schaefer et al.’s: fear, anger, sadness, disgust, amusement, tenderness, neutral). We further call these the ‘self-labels’. Note that our participants were not informed about the video clips’ standard labels. Only in the case of ‘anger’ the self-labels were not univocal as 16 participants reported ‘disgust’ instead (‘Sleepers’, i.e., a video about child abuse). To summarize, we have 3 labels (Table 1):

- standard label: the emotional category of each video clip, taken from Schaefer et al.;
- self-label: the emotional category of each video clip selected by our participants from the list of standard labels used by Schaefer et al.;
- self-reported emotional affect scores of each video, also taken from Schaefer et al.

Unless noted otherwise, we will use the self-labels for labeling the entropy curves.

#### 2.1.2 Participants

The main experiment was performed with 30 healthy volunteers (20 female, 10 male, mean age = 32.48, SD = 15.77, age range 19–70) who master French language (i.e., French as mother tongue or French-Dutch bilinguals). We also recruited 2 non-French speaking volunteers (1 Flemish-Dutch speaking 24 yo male, 1 Armenian speaking 28 yo female). For the experiment with the control videos (with low affect scores), we tested 6 volunteers (2 female, 4 male, mean age = 27, SD = 1.6, age range 25–30). Volunteers were recruited via emails, social media posts, flyers, and billboard announcements. Some were university graduate students (KU Leuven, VUB), often being regular subjects in our EEG experiments, and were paid. No participant had any known neurological or psychiatric disorder. Ethical approval for this study was granted by an independent ethical committee (“Commissie voor Medische Ethiek” of UZ Leuven, our University Hospital). The study was conducted in accordance with the most recent version of the Declaration of Helsinki (2013). Before participating in the experiment, all recruited participants were informed about the goal of the study, what would be their task, and what would be done with the recorded data (privacy), after which they read and signed the informed consent form that was previously approved by the said ethical committee. All EEG recordings were performed between 10/12/2014 and 13/05/2015. The raw scalp EEG recordings are available from https://kuleuven.box.com/v/EntropyEmotion

#### 2.1.3 EEG recording and preprocessing

Participants were tested in a sound-attenuated, darkened room with a constant temperature of 20 degrees, sitting in front of an LCD screen. Each participant’s task was to watch the video clips and report the emotional categories. When viewing the video clips, EEG was recorded^5^ continuously using 32 active electrodes, evenly distributed over the entire scalp (positioning and naming convention following a subset of the extended 10–20 system) using a BioSemi ActiveTwo system (BioSemi, Amsterdam, the Netherlands) as well as an electro-oculogram (EOG) using the set-up of Croft and co-workers [50]. The EEG signals were re-referenced offline from the original common mode sense reference [51] (CMS, positioned next to electrode Pz) to the average of two additional electrodes that were placed on the subject’s mastoids. The duration of the experiment excluding electrode setup was 20 minutes. The EEG signals were filtered using a 4^th^-order Butterworth bandpass filter with range 0.5–30 Hz. The original sampling rate of 2048 Hz was downsampled to 128 Hz (including anti-aliasing) to reduce computational costs. Finally, the EOG signal was utilized for removing eye artifacts following the Revised Artifact-Aligned Averaging (RAAA) procedure described in [50].

#### 2.1.4 Intracranial EEG recording and preprocessing

We also recorded intracranial EEG (iEEG), also termed Electrocorticography (ECoG), from a patient, scheduled for resective surgery, as part of her epileptic seizure treatment, when viewing an emotional and a neutral video clip. The patient was implanted with an intracranial electrode (depth electrode with 10 contacts, contact size 2.4/1.1 mm (overall length/diameter) and 4 mm inter-contact spacing) in the right hippocampus until the amygdala (2 contact points present) and with a subdural electrode grid (4x5 electrodes with 4 mm electrode diameter, 2.3 mm electrode exposure and 10 or 15 mm inter-contact spacing) over the right occipital cortex (S5 Appendix). Recordings were made with a Micromed digital video compatible EEG recording system (Micromed Spa, Mogliano Veneto, Italy). The sampling frequency of the recording was set to 1028 Hz. Offline pre-processing and downsampling were done using the same parameters as for the scalp-recorded EEG, except that a CMS reference per intracranial electrode (10 contacts) and grid (20 contacts) was used. Ethical approval for this study was granted by an independent ethical committee (“Commissie voor Medische Ethiek” of UZ Gent) and conducted in accordance with the most recent version of the Declaration of Helsinki (2013). Before participating in the experiment, the patient was informed about the goal of the study, what would be her task, and what would be done with the recorded data (privacy), after which she read and signed the informed consent form that was previously approved by the said ethical committee. The recordings were done on 29/02/2016.

### 2.2 Methods

#### 2.2.1 Sample entropy

We compare 3 entropy-based methods. The first one is Sample Entropy (SE) [52]. It corresponds to the conditional probability that two sequences that are similar to each other for *m* consecutive data points (samples), within tolerance level *r*, and remain similar when one more data point to each sequence is added. Formally, *SE* is expressed as follows:

$$SE(r,m,N)=-log\frac{A^{m+1}(r)}{B^{m}(r)}$$

where *B*^*m*^(*r*) is the probability that the similarity between two sequences of length *m* obeys the tolerance level *r*, *A*^*m*+1^ (*r*) the probability that the similarity between the same two sequences but now extended with one data point, thus of length *m* + 1, also obeys *r*, and *N* the number of sequences. The tolerance level *r* is usually set to a percentage of the standard deviation of the normalized data; for our case we selected 15% [31].

In order to estimate sample entropy for a multivariate case (MSE), the sequences are formulated as follows. Recalling multivariate embedding theory [53], for *d*-variate time series ${\left\{x_{k,i}\right\}}_{i=1}^{N},k=1,2,\ldots ,d$, the multivariate embedded sequence (so-called a composite delay vector) can be constructed as:

$$X_{m}(i)\;=\;[x_{1,i},\;x_{1,i+\tau_{1}},\;\ldots ,\;x_{1,i+(m_{1}-1)\tau_{1}},\;x_{2,i},\;x_{2,i+\tau_{2}},\;\ldots ,\;x_{2,i+(m_{2}-1)\tau_{2}},,\;x_{p,i},\;x_{d,i+\tau_{d}},\;\ldots ,\;x_{d,i+(m_{p}-1)\tau_{d}}]$$

where $X_{m}(i)\in R^{dm},\;m=\sum_{k=1}^{d}m_{k}$, $[m_{1},m_{2},\ldots ,m_{d}]\in R^{d}$ is the embedding vector and ***τ*** = [*τ*_1_, *τ*_2_, …, *τ*_*d*_] the time lag vector. The above sample entropy definition is then applied to the composite delay vector.

In our case, we have *d* electrode channels, *n* the number of samples or data points in a sequence (further called ‘snippet’ as its length is small in practice), and *N* the number of sequences. The computational complexity of MSE for multichannel EEG recordings is qubic: *ϑ*(*dNn* + *dn*).

#### 2.2.2 Rényi’s entropy

Consider a discrete random variable *x* that adopts *n* values with probabilities *p*_1_, *p*_2_, …, *p*_*n*._ When the *k*-th value delivers *I*_*k*_ bits of information, then the total amount of information becomes the so-called Shannon’s entropy:

$$H(p)=\sum_{k\;=\;1}^{n}p_{k}I_{k}.$$ (1)

In (1), a linear averaging operator is assumed however, in general, for any monotonic function *g(x)* with an inverse *g*^-1^(*x*), the general mean associated with *g*(*x*) for a set of real values {*x*_*k*_, *k* = 1, …, *n*} with probabilities {*p*_*k*_, *k* = 1, …, *n*} can be written as:

$$g^{-1}(\sum_{k\;=\;1}^{n}p_{k}g(x_{k})).$$

Hence, using the general mean, the total amount of information will be:

$$H(p)=g^{-1}(\sum_{k=1}^{n}\left(p_{k}{g(I}_{k}\right))$$ (2)

with *g*(*x*) a Kolmogorov-Nagumo invertible function. Rényi proved that, when the criterion of additivity of independent events applies to (2), then the range of usable functions *g*(*x*) is dramatically restricted. There are two possible classes:

- *g*(*x*) = *c*, where *c* is a constant, so the general mean becomes linear and Shannon entropy (1) is obtained;
- *g*(*x*) = *c*2^(1−*α*)^^*x*^, which implies that the entropy definition becomes:
  $$H_{\alpha }\left(p\right)=\frac{1}{1-\alpha }{\text{log}}_{2}\left(\Sigma_{k=1}^{n}p_{k}^{\alpha }\right),\;\alpha \neq 1,\;\;\alpha \geq 0$$ (3)
  which is called Rényi’s information measure of order *α*.

We are particularly interested in the case where *α* = 2, further called Rényi’s quadratic entropy (RQE), as the term between brackets in (3) corresponds to the expected value of the probability density function (PDF). We wish to estimate RQE directly from the sampled signal using a Gaussian kernel for Parzen’s density estimate:

$${\hat{p}}_{X}(x)=\frac{1}{n\sigma }\sum_{i\;=\;1}^{n}k(\frac{x-x_{i}}{\sigma }),$$

with *N* the number of samples and *σ* the kernel size or bandwith parameter. Hence, we obtain:

$$H_{2}(x)=-{log}_{2}({IP}_{2}(x))$$

$${IP}_{2,\sigma }=\frac{1}{n^{2}}\sum_{i\;=\;1}^{n}\sum_{j\;=\;1}^{n}k(\frac{x_{i}-x_{j}}{\sigma }).$$

where IP is the information potential[54]. Note that, for the multivariate case, the average of the univariate kernels is taken. For ease of reference, we will call it multivariate RQE (MRQE). The bandwidth *σ* is a free parameter that can be chosen according to Silverman’s rule:

$$\sigma =\sigma_{X}{\left(4n^{-1}{\left(2d+1\right)}^{-1}\right)}^{\frac{1}{\left(d+4\right)}},$$

where *n* is the number of data points in a snippet, *d* the data dimensionality (number of electrodes), and *σ*_*X*_ the standard deviation.

The computational complexity of MRQE is quadratic: *ϑ*(*dn* + *n*).

#### 2.2.3 Kernel-based Shannon entropy

As a third entropy-based method, we consider Shannon entropy (ShE):

$$S=-\sum_{k}p_{k}\text{log}p_{k}$$

where *p*_*k*_ is a probability density function (PDF). As in the case of RQE, we estimate the PDF directly from the samples using a Gaussian kernel for Parzen’s density estimate:

$${\hat{p}}_{X}(x)=\frac{1}{N\sigma }\sum_{i\;=\;1}^{n}k(\frac{x-x_{i}}{\sigma }),$$

where *n*is the number of data points in a snippet and *σ* the kernel size or bandwidth parameter. Similarly to RQE, we develop the multivariate case with the Shannon entropy (MShE). The computational complexity of MShE is quadratic as well:*ϑ*(*dn* + *n*).

#### 2.2.4 Multivariate Empirical Mode Decomposition (MEMD)

With Empirical Mode Decomposition (EMD) a signal of length *M* data points is split into a *l* = *log*_2_*M* narrow-band, amplitude/frequency modulated components called Intrinsic Mode Functions (IMFs) [55]:

$$Signal={IMF}_{1}+\;{IMF}_{2}+\;\ldots +\;{IMF}_{l}.$$

IMF_1_ corresponds to the highest frequency component and the subsequent *IMFs* to lower, more narrow-banded frequency components. The last component, *IMF_n_*, is the trend in the signal and is usually omitted from further analysis. The multivariate extension of EMD (MEMD) [31] aligns similar frequency bands of multiple channels, thus, providing an assessment of their possible interdependence (mode alignment property).

#### 2.2.5 MEMD-enhanced multiscale, multivariate entropy

There are at least two ways to compute a multiscale version of entropy when using MEMD: we can compute multivariate entropy (MSE, MRQE or MShE) for each scale individually (IMFs) or for their accumulated scales (cumulative IMFs, CIMFs). We explain the algorithm for the CIMF case:

1. obtain the IMFs for each subject’s entire recording length of each video clip (*M* data points, with *M* in our case between 30000 and 52000, depending on video clip length) by applying the MEMD method to a given number *d* of EEG electrodes;
2. accumulate the IMFs one by one, starting with the first one, CIMF_1_ = IMF_1_, and then add the second IMF to the first, CIMF_2_ = IMF_1_ + IMF_2_, and so on, until all IMFs are added;
3. for each CIMF, calculate multivariate entropy (*d* electrodes) for each non-overlapping snippet of a prior defined EEG signal track and take the average over all those *N* snippets (MSE) or calculate the univariate entropy per electrode for those snippets and take the average over all *d* electrodes (MRQE, MShE);
4. plot the entropy estimates as a function of CIMF_1_,CIMF_2_, … to obtain the MEMD-enhanced multiscale, multivariate entropy curve of the targeted EEG signal track (i.e., MEMD-enhanced MMSE, MMRQE, MMShE)

Note that the total number of IMFs is log_2_ *M* [55] but the used algorithm selects by itself the number of IMFs in a data-driven, subject-dependent way (in our case, between 15 and 17 IMFs), hence, for clarity’s sake, we decided to show 15 IMFs for all subjects. Both operations imply that the original signal is only approximated by CIMF_n_, so their entropies could be different.

For the interested reader, the Matlab code for the multiscale MSE algorithm can we found at http://www.commsp.ee.ic.ac.uk/~mandic/research/Complexity_Stuff.htm, and the Matlab code for the multivariate EMD at http://www.commsp.ee.ic.ac.uk/~mandic/research/emd.htm

#### 2.2.6 Scalp space projection of intracranial recordings

In order to explore the relation between scalp- and intracranial recordings, in response to emotion-evoking and neutral video clips, we performed a source reconstruction analysis with the Brainstorm toolbox as it can work directly with intracranial electrodes. We started with the patient’s post-implantation MRI headscan to define the standard Montreal Neurological Institute (MNI) stereotactic coordinates of the depth electrode’s contact points in the amygdala-hippocampal complex and the electrode positions of the grid covering the occipital cortex. Then, we used the patient’s MRI scan before implantation to extract the cortex envelopes from the MRI scan (inner and outer skull, scalp surface and cortex) using the Brainsuite software[56]. Fiducial points were selected manually. For the forward model, Boundary Element Model (BEM) surfaces were created using 15000 dipoles on the entire MRI volume so as to include deep sources, using the OpenMEEG BEM model [57]. For the inverse modeling, as there was no resting state data available, the identity matrix was selected for the noise covariance, and the sLORETA algorithm used for distributed source modeling [58].

The depth electrode had 10 contact points but, based on anatomical grounds, the first 2 contact points were considered to be in the amygdala. However, as their recordings did not display a linear relation (no obvious Pearson correlation, see S4 Appendix), but instead the recordings of the second contact point seemed to correlate with those of contact points 3 to 6, we only used electrode 1 to simulate data from that contact point to see how its projects back on scalp space. In order to obtain a sizeable estimate of amygdala activation, we assumed that 1 cm^3^ of amygdala tissue, in proportion to the entire head volume, corresponds to about 27 amygdala dipoles out of 15000 in total. We put all values of the headmodel to zero, except for the said 27 dipoles, which we filled with contact point 1’s recordings. As the headmodel for the entire MRI volume is not constrained in orientation and orientation, but needs to be defined for our simulations, we chose four possible orientations for simulating our deep source—the X-, Y-, and Z-directions, and also the equally mixed version, with an equal weight in each direction—, and projected the result on the entire scalp.

Next, we wanted examine whether the sources that generated the intracranial recordings of the electrode grid covering the occipital lobe also contribute to the frontal scalp recordings. For this, we first solved the inverse problem, thus starting from the 20 electrode grid recordings, and then addressed the forward problem to see how these sources show up in the frontal part of the scalp.

## 3. Results

### 3.1 Discriminability of emotional categories

**Fig 1:**
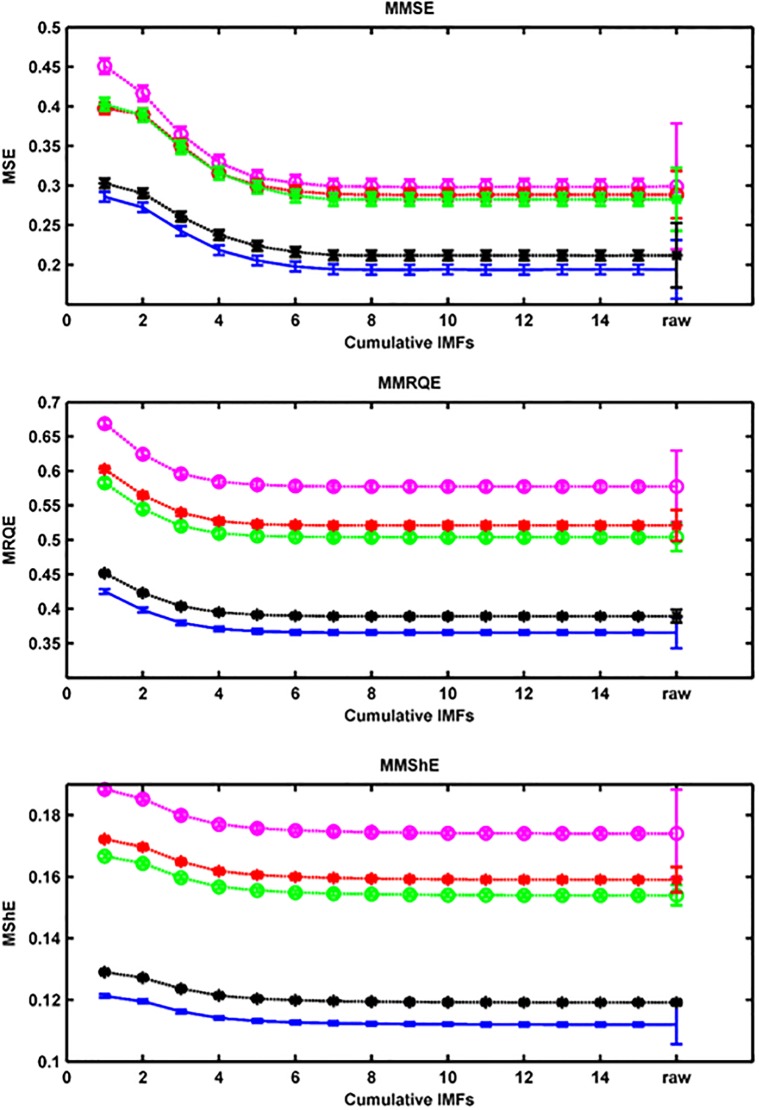
MEMD-enhanced MMSE, MMRQE and MMShE curves, main experiment. Curves are labeled in terms of our 30 participants’ self-reports (self-labels) and corresponding uniscale entropy values for the original EEG recordings (cf., horizontal axis tick labeled ‘raw’). Error bars are standard errors of average MMSE, MMRQE and MMShE values per subject. Results shown for 100 ms snippet length. Color convention: purple = anger, red = amusement, green = disgust, black = tenderness, blue = sadness.

**Fig 2:**
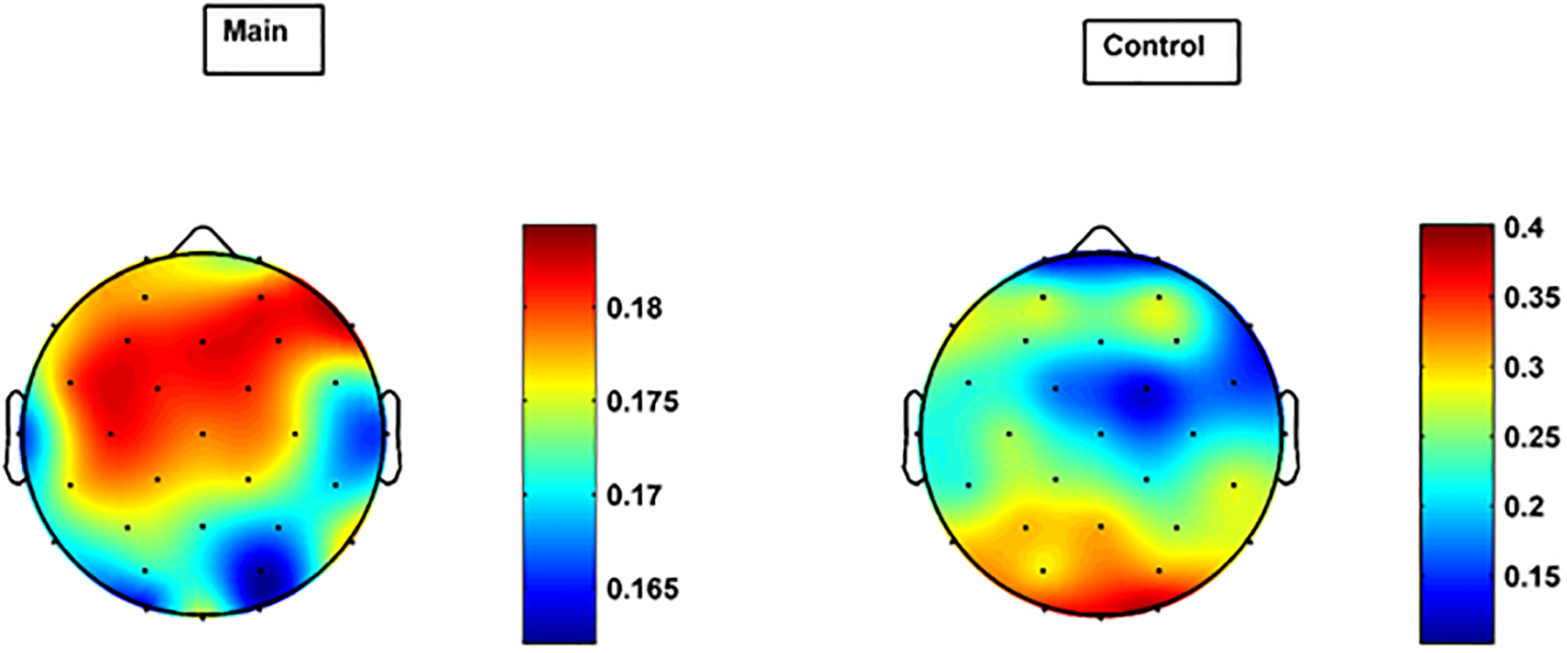
Scalp distribution of Krippendorf's alpha coefficient for the main and control group. The coefficient shows the degree to which the participants’ RQEs for CIMF_6_ are in agreement.

MEMD-enhanced multiscale, multivariate entropy estimates of the preprocessed scalp EEG recordings were computed only over the last 100s of each video clip, as the video clips were construed by Schaefer and co-workers to have their climax at the very end (see also section 3.5). For the sake of comparison, in the same way as in, we considered electrodes F3 and F4 (mid-frontal area) (*d* = 2) and also took *N* = 1000 non-overlapping snippets of length 100 ms (*m* = 12), and averaged the entropy estimates across snippets of each participant. The MEMD-enhanced MMSE, -MMRQE and -MMShE curves for all 30 participants, grouped by their self-reported emotional categories (see Table 1), are shown in Fig 1. We observe that the complexity curves of the three entropies are similar with similar discriminabilities between emotional states. We also observe that the curve labeled ‘sadness’ has the lowest entropy values—note it also has the lowest affect score of all video clips used—, thus, confirming Aftanas and co-workers’ assertion [21]. However, when assessing the effect of snippet length, we observe that the MEMD-enhanced MMRQE curves are less sensitive to the choice of snippet length compared to those of the other 2 approaches (see S1 Appendix). We also calculated the entropies of the original (uniscale) EEG. We observe that the uniscale case exhibits a much lower discriminability (factually only 2 groups of plots remain), clearly showing the advantage of the multiscale approach. We also calculated the MEMD-enhanced MMRQE curves for the 2 non-French speaking participants (S6 Appendix.). The curves are now factually indistinguishable except for the ‘Life is beautiful’ video clip (self-labeled as tenderness). Hence, the physical parameters of the video clips are not explaining the discriminability of the MMRQE curves (see also the low interrater agreement of the occipital and auditory cortex responses in Fig 2, left panel, discussed further).

We also computed the MEMD-enhanced MMRQE curves of the 6 participants to the control experiment (see S7 Appendix). Note that the 4 video clips were not taken from the top ten list of affect scores (Table 1). The individual curves are much less discriminable and their ranking—in terms of self-labels—is now subject-dependent.

As the peak frequency of IMF_*i*_ is given by $\frac{sampling\;rate}{2^{i+1}}$ [55], given the *sampling rate* of 128 Hz, the frequency range spanned by the first 6 IMFs is from 1 Hz to 32 Hz and this corresponds to the bandwidth of our Butterworth filter implemented in the preprocessing (0.5–30 Hz). This also explains the asymptotic behavior of our entropy curve results, whence, we will further focus on CIMF_6_. When applying a linear mixed model [59] to the CIMF_6_ results of MMRQE, with self-label, gender and age as fixed effects, and subject as random effect, we found that self-label is significant (< 0.0001), whereas age (p = 0.083) and gender are not (p = 0.68).

For comparison’s sake, we also considered the case where entropy is plotted as a function of IMF index, thus, by considering separate scales. The result is shown in S2 Appendix.: we observe a much lower discriminability between emotional states compared to the cumulative case (CIMF).

Finally, as the computational complexity of MMRQE is quadratic and that of MMSE cubic the former is also advantageous for computational efficiency reasons.

### 3.2 Discriminability of multiscale RQE per electrode

**Fig 3:**
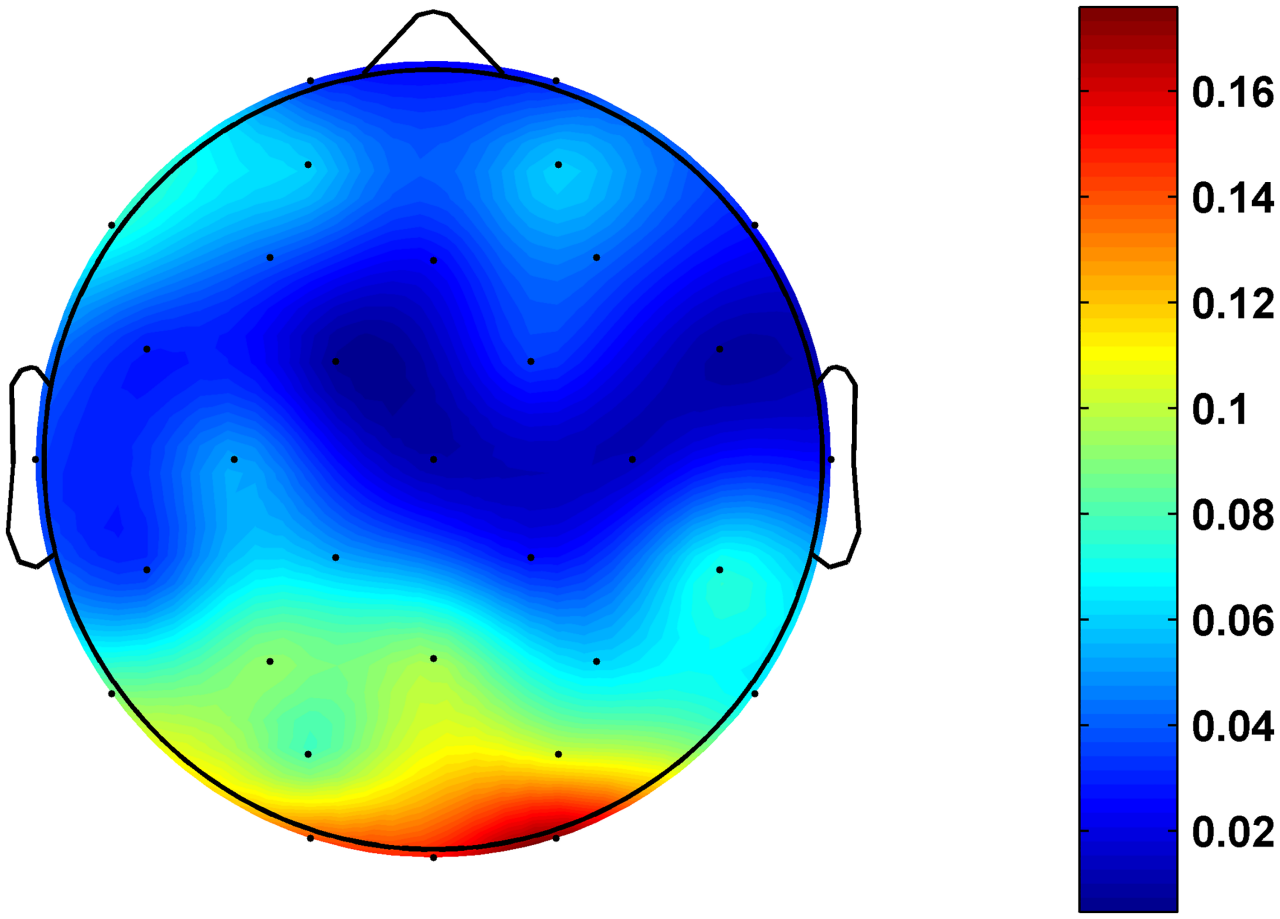
Scalp distribution of *p* values of the difference in alpha coefficient between main and control groups.

We statistically assessed to what extent the RQEs per self-reported emotional category are in agreement across subjects (inter-rater reliability, inter-rater agreement). We used the so-called interval version of Krippendorf's alpha statistic (using the *kriAlpha* function in Matlab) for our 30 participants (main experiment), given their 5 self-reported emotional categories (self-labels), and determined the scalp distribution (per electrode, thus, univariate) of the alpha statistic of the RQEs and restricted ourselves to CIMF_6_, to simplify the comparison. The results are shown in Fig 2 (left panel) when using 100ms snippets on the last 100s of each video, and when computing the EMD for all 32 electrodes jointly (MEMD) and retaining CIMF_6_. We observe that the frontal region of the scalp has the highest alpha coefficient with much smaller values for the occipital and auditory cortices. For comparison’s sake, we also computed the scalp distribution of the alpha coefficient for the original (uniscale) EEG data (see S3 Appendix: the coefficients now turn out to be negative, implying a disconcordance in the RQE values between subjects). In order to assess the statistical significance of our results, we compared the alpha coefficient of each electrode of the main group (30 subjects) with that of the corresponding electrode of the control group (6 subjects). Hereto, we determined a distribution of the alpha coefficient of the control group using a simple block bootstrap strategy (Matlab’s *bootstrp* function) in which the alpha coefficient is computed for 1000 subsets of 5 subjects (random sampling with replacement). The result for the control group is shown in Fig 2 (right panel). The scalp plot of the *p* values resulting from a simple t-test is shown in Fig 3. One observes that the most discriminative electrodes are F3, FC1, FC5, T7, CP5, CP2, C4, T8, FC6, F4, F8 (using a 5% significance level).

**Fig 4:**
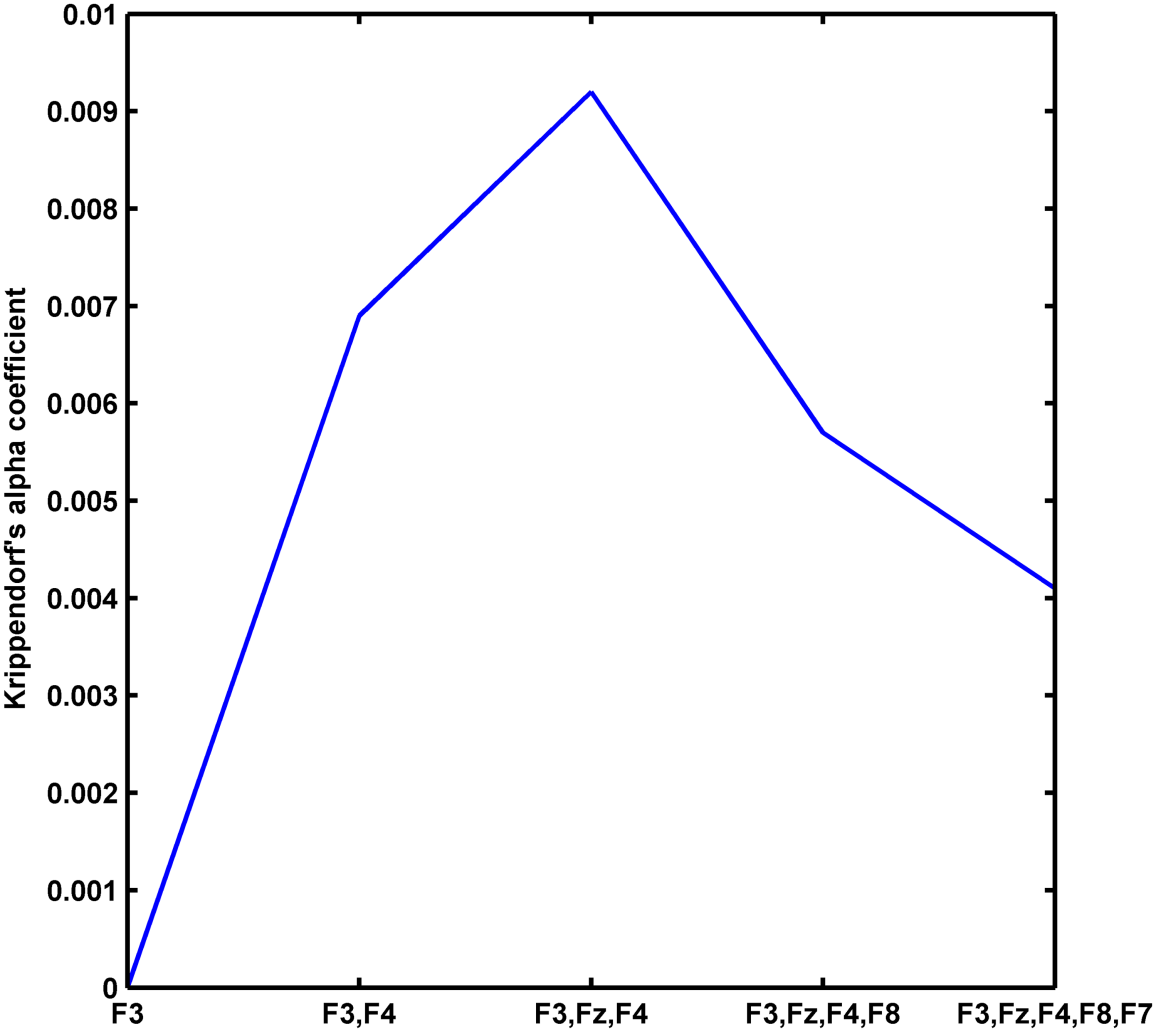
Clustering electrodes based on alpha statistics (main group).

In order to identify which subset of electrodes best discriminate between the 5 self-reported emotional categories, we conducted a ‘greedy’ search on the alpha coefficients of the main group (30 participants) computed from the last 100 seconds of each movie. We started the search from F3, the most significant electrode in Fig 2 (left panel), computed CIMF_6_ for this electrode only (to avoid effects from other electrodes), and plotted its alpha coefficient based on the main group’s RQE. Then, we added the next most significant channel, F4, compute CIMF_6_ for F3-F4, and plotted the alpha coefficient based on multivariate RQE (channels F3-F4, thus d = 2), and so on (Fig 4). One observes that F3-F4-Fz show best discriminability. This confirms that, at least for our case, emotion discrimination can be achieved using only 3 mid-frontal channels, which also reduces the computational cost for the MEMD-enhanced MMRQE algorithm (remember that the algorithm’s complexity scales with number of channels).

### 3.3 Temporal evolution of multiscale RQE discriminability

**Fig 5:**
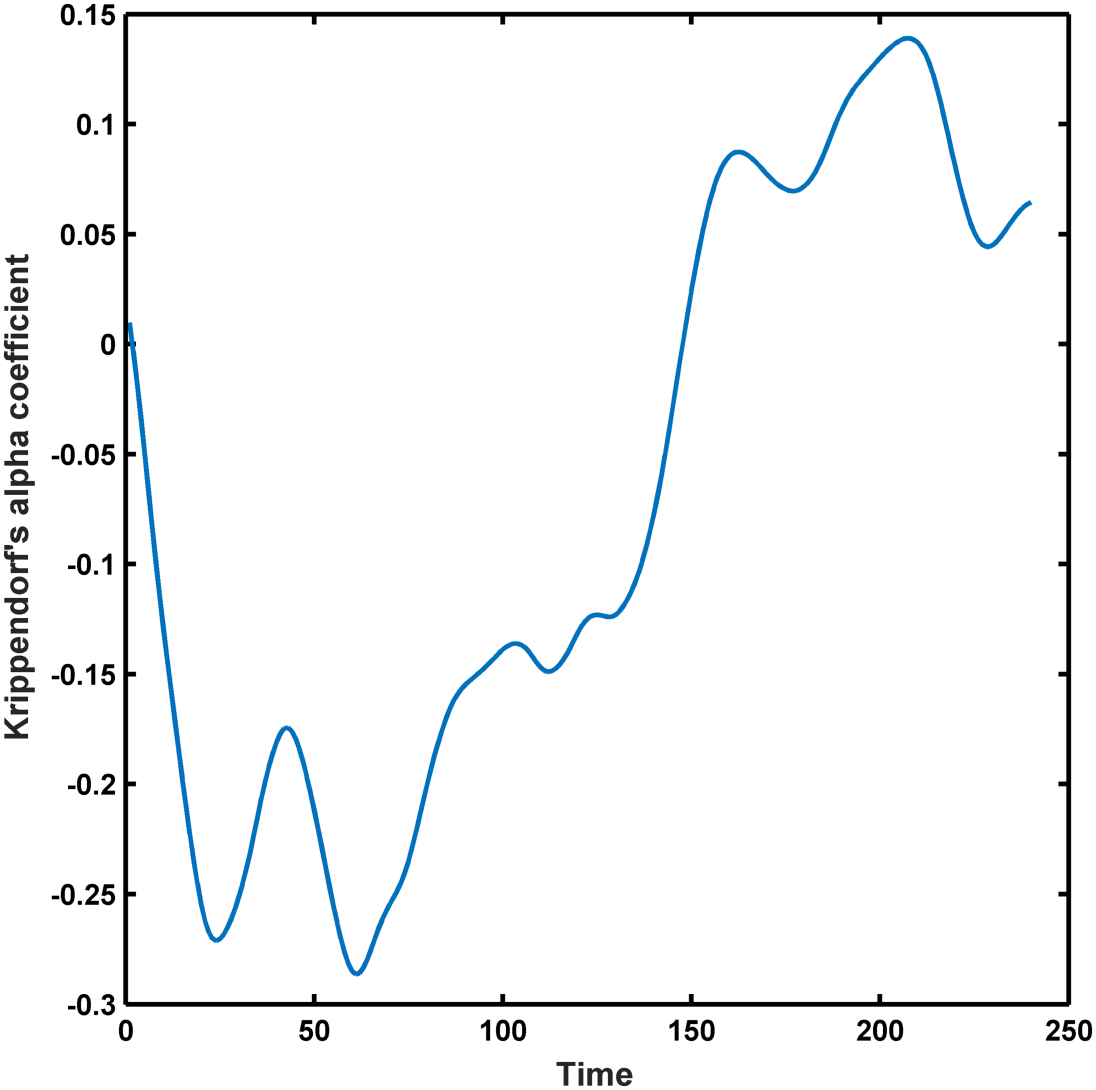
Evolution of Krippendorf’s alpha statistic of MRQE for CIMF_6_ over entire video clips, plotted for mid-frontal electrodes.

We also considered the temporal evolution of Krippendorf’s alpha coefficient of the main group but now using the entire length of each video clip. We used 100ms snippets, the 3 mid-frontal (F3, F4, Fz) electrodes selected by our clustering analysis for MEMD calculation (Fig 4), and compute the multivariate RQE (MRQE, *d* = 3) for CIMF_6_. We observe from Fig 5 that the alpha coefficient, and whence the discriminability across participants, increases and remains relatively stable towards the end of the video clip. This confirms our motivation to focus on the last 100s of the EEG recordings.

### 3.4 Multiscale RQE of intracranial EEG

**Fig 6:**
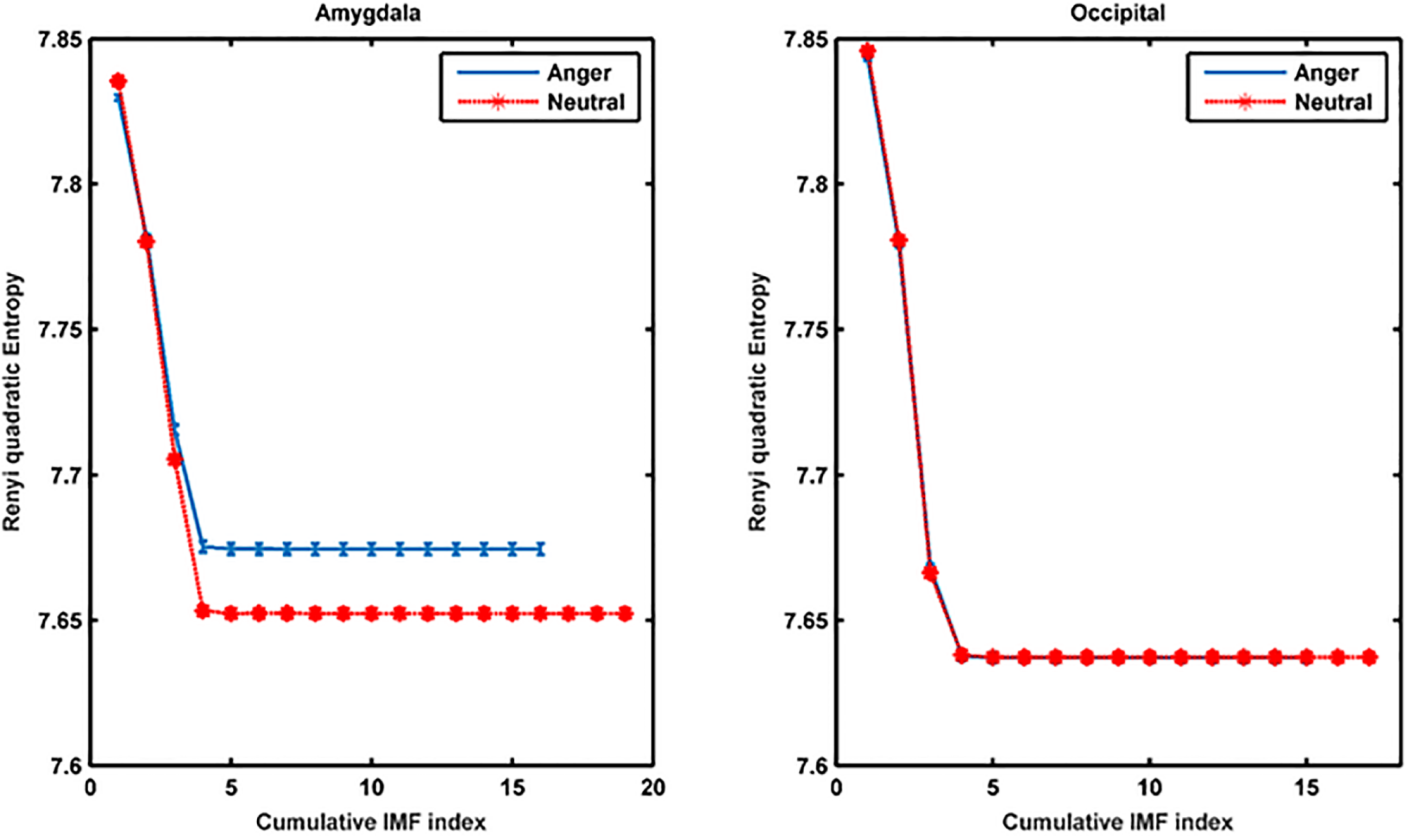
MEMD-enhanced MMRQE curves for intracranial EEG recordings. Shown are the results for the (a) amygdala- and (b) occipital implants in a patient viewing emotional and neutral video clips (blue vs. red labeled curves). Error bars correspond to standard errors of individual snippet MMRQEs.

We also computed the MEMD-enhanced MMRQE for the intracranial EEG recordings in the amygdala (2 contact points) and over the occipital area (20 contact points) when the patient viewed an emotional- (fragment from ‘Schindler’s list’, self-labeled by the patient as ‘anger’) and a neutral (weather forecast, self-labeled as ‘neutral’) video clip (Fig 6). As in the scalp EEG case, we selected the last 100s track of each video clip, determined the MEMD for the 2 amygdala contact points (depth electrode) and separately for the 20 occipital contact points (subdural electrode grid), and finally plotted the multiscale, multivariate RQE (MMRQE) using non-overlapping 100ms snippets. We observe that the MMRQE curves for the amygdala are well separable compared to those of the occipital area (Fig 6, left vs. right panel).

### 3.5 Temporal evolution of multiscale RQE for joint scalp/intracranial EEG

**Fig 7:**
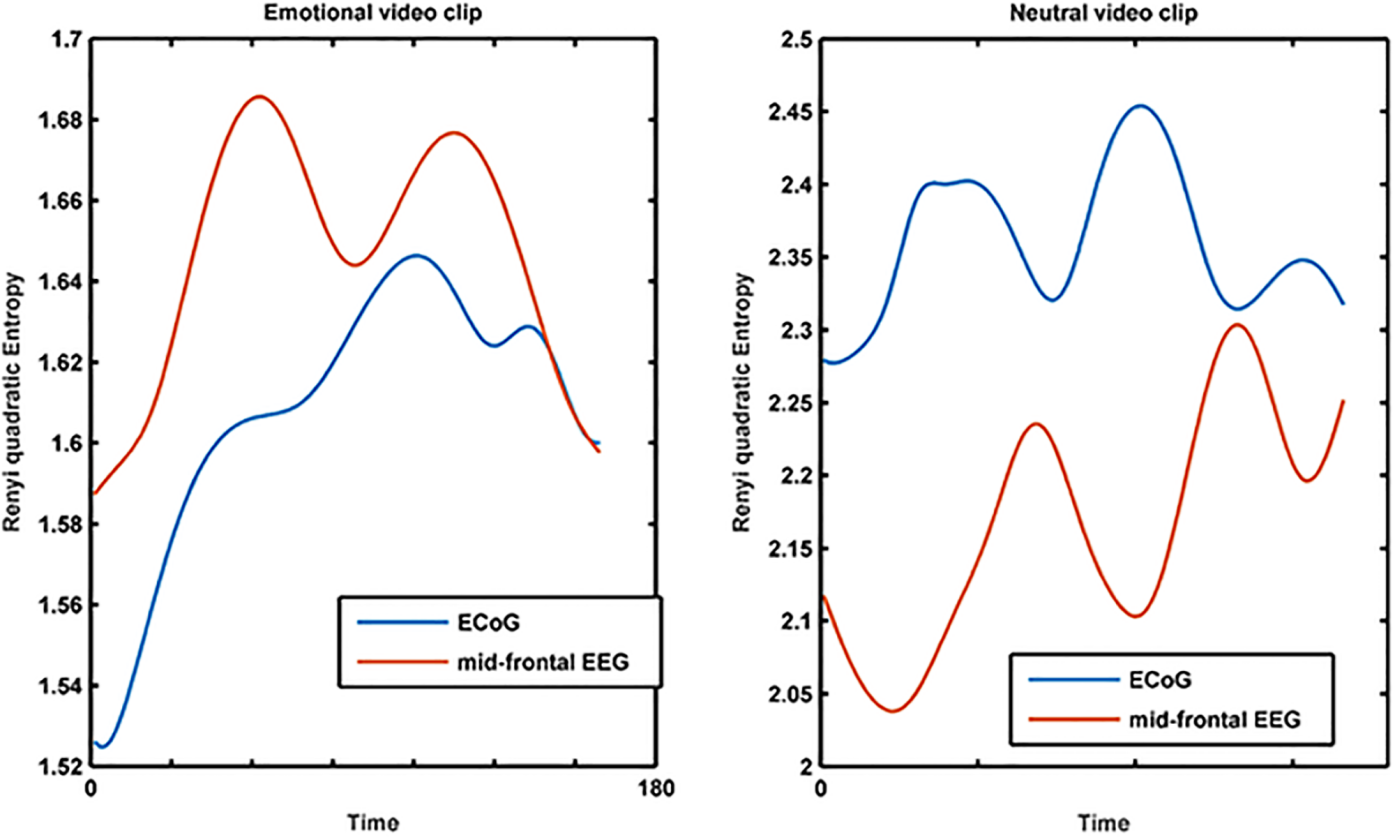
Temporal evolution of multivariate RQE. Shown are the results for CIMF_6_ for amygdala iEEG and mid-frontal scalp EEG (F3, F4, Fz electrodes) in response to emotional and neutral video clips.

Finally, we computed MRQE for CIMF_6_ using the joint scalp (mid-frontal) EEG and intracranial EEG (amygdala) recorded when viewing both video clips (emotional and neutral). Note that the MEMD is computed with reference to the listed electrodes in each case. The results are shown in Fig 7. We observe that, for the ‘anger’ video, the MRQE curves of the intracranial EEG (amygdala) and mid-frontal scalp EEG seem to converge and even overlap at the end of the videos (Fig 7, left panel). In order to statistically assess this observation, we modeled the temporal evolution of both MRQEs for 8 successive, non-overlapping time intervals where each interval consists of 20 successive recording samples (interval between 2 successive samples is 1s). Then, for each recording, we adopted a linear mixed model approach [59,60] with MRQE as continuous outcome and time intervals as fixed effects:

$${MRQE}_{i}=\alpha +a_{i}+{(\beta }_{1}+b_{i})*{time\_interval}_{1i}+\cdots +(\beta_{7}+b_{i})*{time\_interval}_{7i}+\varepsilon_{i}$$

where subscript *i* refers to the modeled recording, *α* the overall intercept, *β*_1_ … *β*_7_ the overall time interval-specific slopes, the latent variables $(a_{i},b_{i})\sim N(0,(\begin{array}{cc} \sigma_{1}^{2} & 0\\ 0 & \sigma_{2}^{2} \end{array}))\;$ random intercept and -slope, and *ε*_*ij*_~*N*(0, *σ*^2^) random noise. To correct for possible EEG recording-specific differences, random intercept and random slope were used. Finally, we tested the hypothesis that the MRQEs of both signals (scalp and intracranial) at a given time interval were from the same distribution. For all time intervals, expect the two last ones (40 ms in total), the hypothesis is rejected, hence the MRQE curves indeed become indistinguishable at the end of the ‘anger’ video. Note that this also corresponds to the time range producing best discriminability (alpha coefficient) of the main group’s scalp EEG recordings (section 3.3), where the crux of the video unfolds. On the contrary, no such overlap is observed for the ‘neutral’ video (Fig 7, right panel). All analyses were performed using SAS, release 9.4.

**Fig 8:**
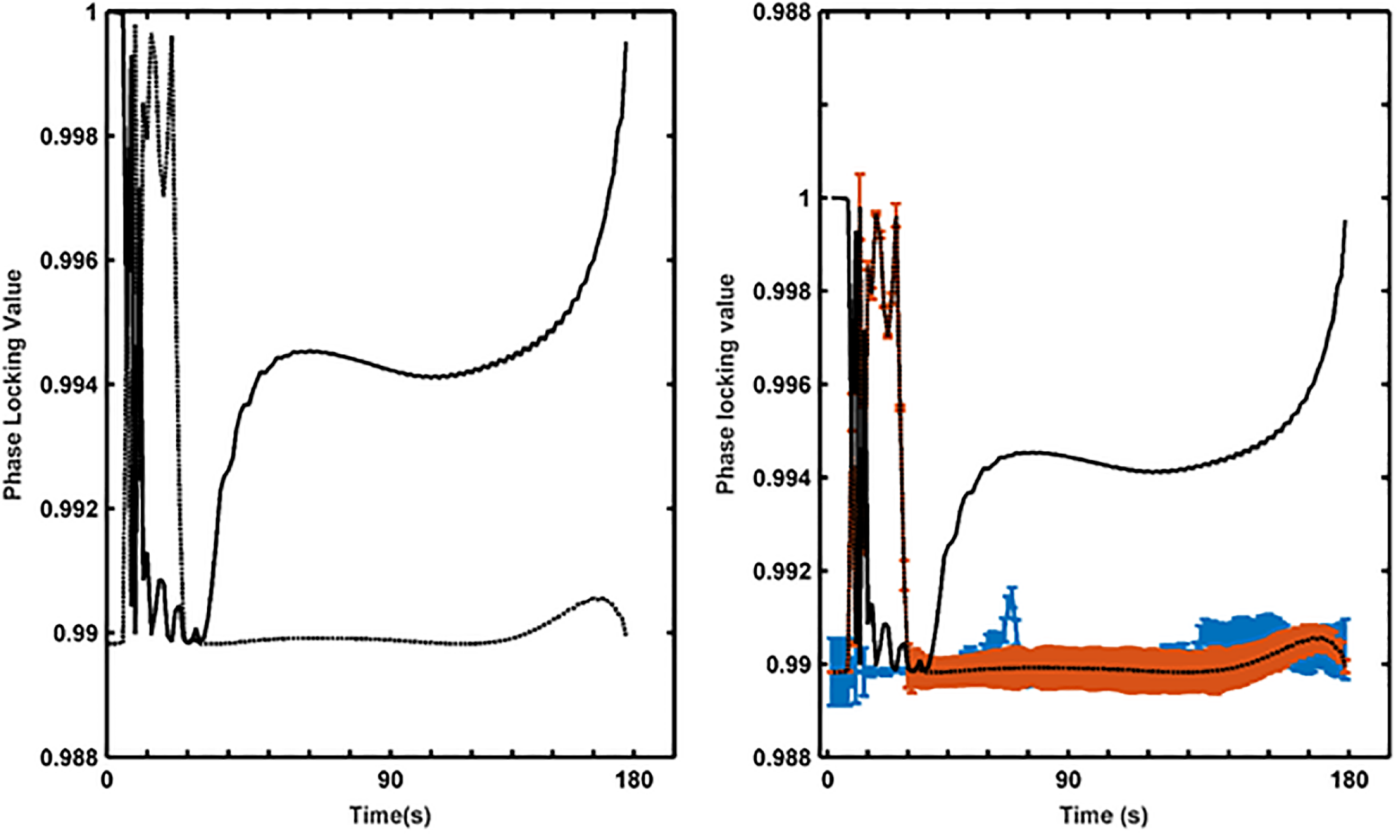
Phase synchrony between mid-frontal EEG and amygdala. Shown are the results for the (a) emotional (‘anger’) and neutral video clips (left) and for the corresponding surrogate signal distributions (right) (red = neural video, blue = emotional video).

In order to gain insight into the origin of the overlap between the two MRQE curves, we analyzed phase synchrony between the mid-frontal scalp EEG and intracranial amygdala signals, using the phase locking statistics (PLS) technique introduced by Lachaux and co-workers [61]. The results are shown in Fig 8 (left panel). After a transitional period, at the onset of both videos, phase synchrony of the emotional video (‘anger’) increases sharply towards the end, which is not the case for the neutral video as phase synchrony stays constant. As these results are from only one subject, there is not enough statistical power to decide whether the difference between the two curves and between the low and high synchrony values of the emotional video are significant. We therefore applied the surrogate data method called Iterative Amplitude Adjusted Fourier Transformed (IAAFT), originally proposed by Schreiber and Schmitz [62] [63], to the amygdala and mid-frontal recordings of each video. The IAAFT algorithm has ability to generate surrogate signals with identical amplitude distributions and approximately identical amplitude spectra while the cross-correlation between the original signals is destroyed. We used Matlab’s Chaotic Systems Toolbox (https://nl.mathworks.com/matlabcentral/fileexchange/1597-chaotic-systems-toolbox/content/IAAFT.m) with c = 10, maxiter = 1000, frequency range 0.05–30 Hz, and trial length 200ms.

The results are illustrated in Fig 8 (right panel). It can be seen that the increasing phase synchrony of the emotional video is significantly different from its surrogate distribution whereas the phase synchrony of the neutral video is indistinguishable from its surrogate distribution. The high value of the phase synchrony at the end of the emotional video supports the similarity in MRQE values between the scalp and intracranial recordings (Fig 7): this means that these recordings exhibit strong phase synchrony and share similar degrees of complexity near the end of the emotional video.

Finally, using Brainstorm, we simulated for each of the 4 different amygdala source orientations (see Methods section 2.2.6) the EEG signal (128 channel full scalp configuration) in response to the available amygdala recordings of both video clips. As above, we computed phase synchrony between simulated and actually recorded mid-frontal (F3,Fz,F4) EEG and again applied the IAAFT algorithm to generate surrogates. The results are shown in S8 Appendix for the case where the amygdala dipole orientations are equally weighted in X-, Y-, and Z-direction (“mixed” case, see Methods section 2.2.6); the results for the X-, Y-, and Z-oriented dipoles are comparable. We observe that phase synchrony behaves quite similarly to Fig 8. The phase synchrony result in S8 Appendix supports our hypothesis that, at least for the last part of the emotional video, amygdala could indeed be contributing to the synchrony observed in our mid-frontal scalp recordings, but we hasten to add that other sources (which we did not model here) could be contributing as well. When repeating the exercise for the sources that evoked the occipital electrode grid response and also simulate their EEG activity on the scalp, we observed no significant phase synchrony levels for the mid-frontal electrodes neither any difference between the neutral and emotional videos. This result is also in line with our complexity results (Fig 8).

## 4. Discussion

Recently, several algorithms were proposed linking EEG signal complexity (in particular entropy) to distinct emotional states. Jie and co-workers [23] performed emotion recognition from univariate, uniscale sample entropy (SE) of prefrontal EEG recordings. However, the EEG recordings of subjects whose emotion self-reports were not in line with the pre-defined ones were excluded from their analysis. In our previous work [34], we used MEMD-enhanced multiscale, multivariate sample entropy (MMSE) to discriminate multiple emotional states from EEG recordings in response to emotion-evoking video clips. Contrary to Jie and co-workers, we used self-reports of emotional category for labeling the signal complexity results. Furthermore, unlike previous studies [64], the emotional state was discriminated even from a single video clip (single-trial design). This strategy was also adopted in the pertinent article but in the context of MEMD-enhanced MMRQE, a multiscale, multivariate version of Rényi’s quadratic entropy (RQE). Albeit the results were similar, the Rényi-based approach revealed two advantages. Firstly, MMRQE’s computational complexity is quadratic whereas MMSE’s [34] cubic, which rendered the Rényi-based approach computationally much more feasible for joint electrode analyses and electrode selection strategies. Secondly, the MMSE-based method is much more sensitive to snippet length as the discriminability of emotional categories quickly degrades with snippet length. For MMRQE we observed a much broader range of usable snippet lengths.

It has been claimed that changes in emotional state (also in relation to dysfunctions) can be discerned from frontal lobe recordings [65,66]. In [67] it was shown that the orbital frontal cortex plays a critical role in cognitive control of emotion (especially in the case of suppressing emotional responses), and activity in this region reflects subsequent appraisal processes related to viewing emotional stimuli (see also [38]). Jie et al. [23] achieved emotion recognition from prefrontal EEG recordings and Tonoyan et al.[34] from mid-frontal recordings. By using Krippendorff’s inter-rater reliability analysis in combination with a greedy search for optimal electrodes, we showed in the pertinent article that the mid-frontal electrodes F3, Fz, F4 scored best in discriminating emotional states (Fig 4) but this does not preclude the contribution of central and temporal electrodes, as reported in other studies [37,44,68]. Interestingly, EEG signals over the occipital and auditory cortices exhibited a much lower discriminability which implies that our results are probably not explainable by sensory or auditory activations elicited when viewing video clips.

The question arises whether EEG signal complexity could be used to ‘label’ arbitrary video clips in terms of emotional states. There are several observations that demote our expectations. Firstly, there is the concern on the labeling side: the same video clip could receive different emotional category reports from different viewers as we observed for the ‘Sleepers’ movie (Table 1). Another concern, maybe the most important one, is that signal complexity is not static but rather evolves when watching the video clip and this affected the discriminability of our signal complexity results in terms of self-reported emotional categories (see Fig 5). However, we also observed that discriminability increased towards the end of the video clips. This is in alignment with the way the video clips were constructed by Schaefer and co-workers: the clips have their climax at the end (roughly the last 100s). We therefore recommend to restrict EEG complexity analysis to the most affective scenes as for those the highest inter-rater agreement can be expected.

How to interpret signal complexity then? One suggestion is to relate it to affect score: the affect scores of the video clips we used are available in [35] and are averages of self-reported affect scores based on 10 positive and 10 negative items (each on a 5 point scale). The video clip ‘City of angels’ showed both a lower affect score (Table 1) and a lower MMRQE curve (Fig 1) than the ‘La cité de la peur’ video clip, which is in alignment with the assertion of Aftanas and co-workers [21] that higher complexity values can be observed for more affective stimuli. However, we could not observe such a relationship for the other video clips. This could be due to the ways the affect scores of [35] (20 item check list per clip) and our emotional self-scores were collected (one emotional category per clip), and whether differences in reported affect scores are significant. (Note that no standard deviations are provided in [35].) Video clips with low affect score seem to be more difficult to discriminate based on signal complexity, as we showed for the control experiment S7 Appendix): albeit the range of the RQE values was similar to that of the main experiment, the MMRQE curves were much less discriminable and their ranking varied across participants. By using video clips with the highest affect scores (cf., 3 of the 4 video clips of the main experiment), or by contrasting highest and lowest affect score video clips (cf., ‘City of angels’ video clip), we could achieve discriminability based on signal complexity (Fig 1). Affect score, thus, seems to play a role in signal complexity but not completely. Another suggestion is to relate signal complexity to emotional arousal as these self-reports are also available in [35]. As shown in our earlier work, and it also pertains to our MMRQE results, the relation between signal complexity and emotional arousal was not significant (using a linear mixed model regression analysis) neither for the whole video clip (since in [35] the self-reports were collected in this way) nor for the last 100 s (‘climax’).

In order to shed light on the significance of scalp EEG signal complexity, we also examined intracranial EEGs from the amygdala recorded in a patient when viewing emotional and neutral video clips. The amygdala is considered to be strongly involved in emotional processing. Guillory and co-workers [69] reviewed 64 invasive studies on human emotion including amygdala [37–46]. Moreover, Sergerie and co-workers [47] showed that the amygdala responds to all visual emotional stimuli regardless of their valence and Aggleton and Mishkin even claimed it is the gateway to sensory emotion [48]. By applying the same MEMD-based MMRQE method to the intracranial recordings, we observed a clear discrimination between the MMRQE curves of the neutral and the emotional movies (labeled by the patient as evoking anger) for the amygdala but not for the occipital area (Fig 6). This result is in agreement with several studies that prove the involvement of amygdala in negative emotion processing. Oya et al. [40] proved that significant changes in gamma power amplitude in amygdala were selectively obtained in response to visual images judged to be aversive but not in response to those that were judged pleasant or neutral. Naccache and co-workers [42] obtained differential ERP responses in amygdala to negative emotional words. We observed that the multiscale RQE computed for the mid-frontal scalp electrodes and the amygdala electrodes overlapped and their phase synchrony reached a maximum at the end of the emotional video where the crux is revealed. On the contrary, no such overlap or phase synchrony was observed for the ‘neutral’ video. This was also observed for the phase synchrony between the original- and reconstructed mid-frontal scalp activations, reconstructed from our amygdala recordings using our head model. In summary, our findings seem to suggest that emotional state discrimination from scalp EEG is more likely to be supported by differences in amygdala activation when in response to emotionally-charged scenes.

## Conclusion

We introduced multiscale, multivariate Rényi quadratic entropy (MMRQE) for analyzing EEG recordings of 30 participants viewing 4 emotionally-charged video clips taken from a validated database [35]. We compared our approach to the multiscale, multivariate version of sample entropy (MMSE), and showed that the results were similar but with the advantage that MMRQE is less computationally intensive and less sensitive to track (snippet) length. We also applied our method to intracranial EEG recordings of the amygdala and observed that the RQEs of the emotional and neutral video clips could be discriminated and that the MMRQE of the emotional video coincided with that of the mid-frontal scalp electrodes at the end of the clip where the climax of the video is revealed. This was also confirmed by the increasing phase synchrony levels between the mid-frontal and amygdala recordings. This provides conclusive evidence of the proposed multiscale entropy-based method in discriminating emotional states.

## Supporting information

S1 Appendix Effect of snippet length. (PDF)

S2 Appendix MMRQE plotted in terms of individual IMFs. (PDF)

S3 Appendix Scalp distribution of original EEG’s alpha coefficient. (PDF)

S4 Appendix Correlation matrix of depth electrode. (PDF)

S5 Appendix Location of subdural electrode grid. (PDF)

S6 Appendix MMRQE results for non-French speaking subjects, main experiment. (PDF)

S7 Appendix MMRQE results, control experiment. (PDF)

S8 Appendix Phase synchrony between simulated and mid-frontal EEG. (PDF)
