# Supplementary material for: Discrimination of emotional states from scalp- and intracranial EEG using multiscale Rényi entropy
Source: PLoS One. 2017 Nov 3;12(11):e0186916. doi: 10.1371/journal.pone.0186916 (PMC5669426; doi:10.1371/journal.pone.0186916)
Supplement: S1 Appendix — (PDF) [file pone.0186916.s001.pdf]

S1 : Effect of snippet length

MMRQE

100ms	200ms	400ms

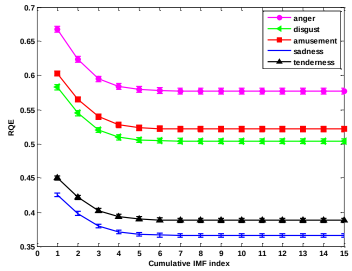

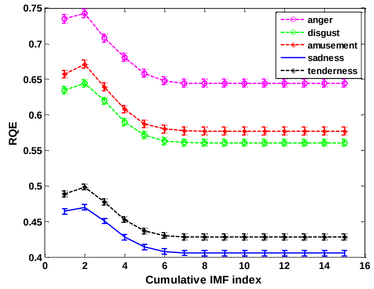

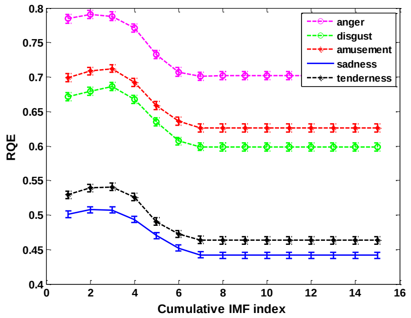

800ms	1sec	10sec

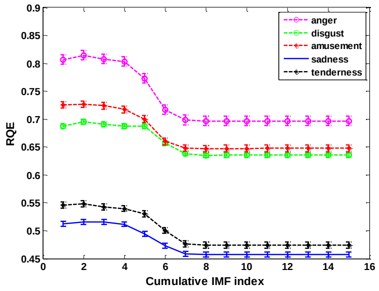

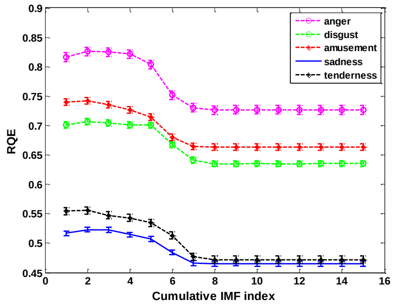

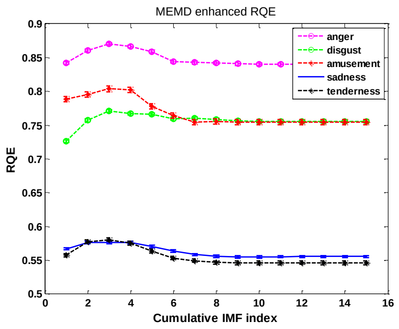

MMSE

100ms	200ms	400ms

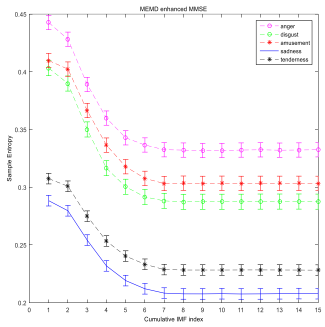

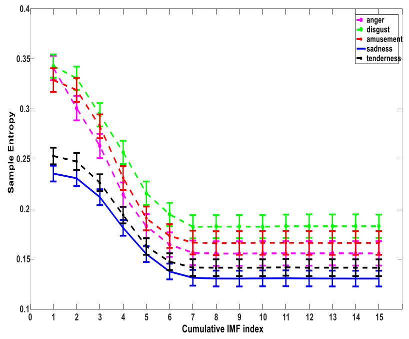

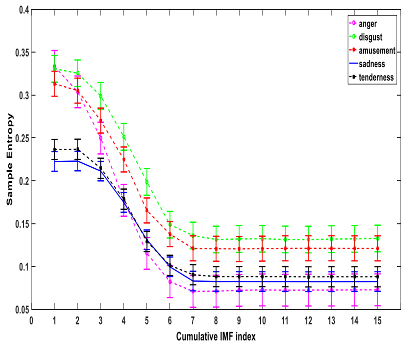

800ms	1sec	10sec

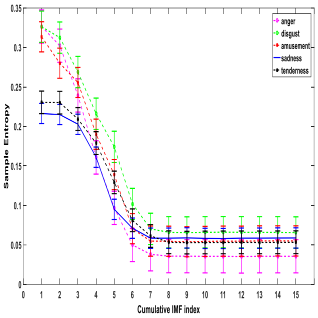

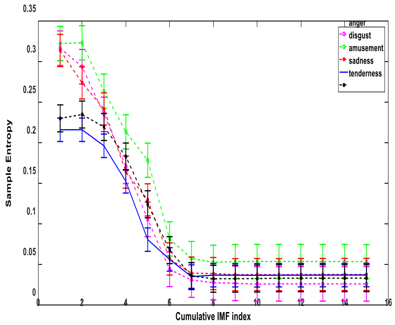

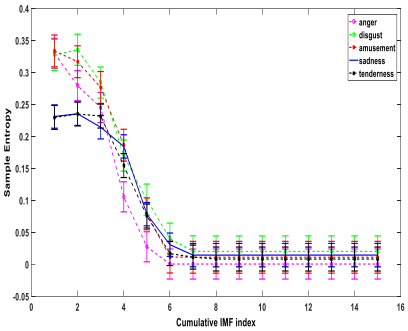

MMShE

100ms

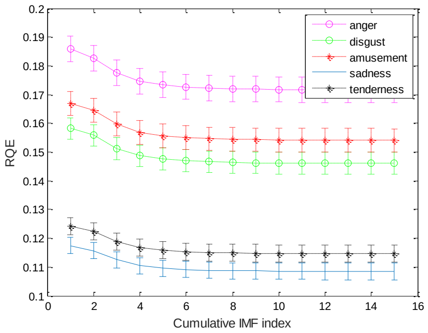

200ms

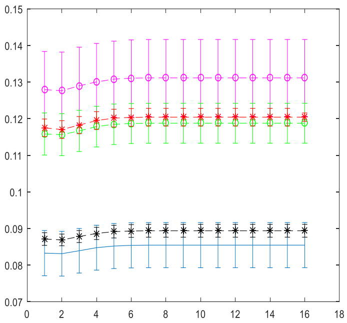

400ms

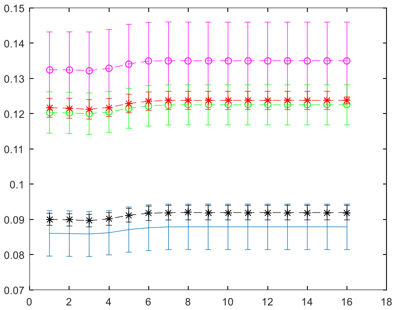

800ms

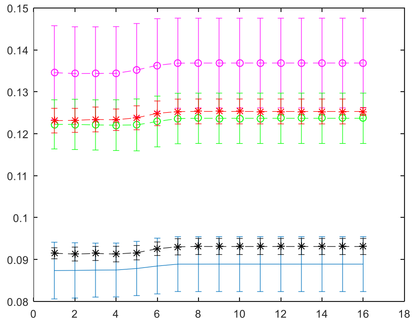

1sec

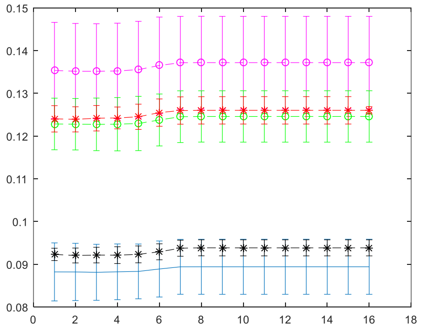

10sec

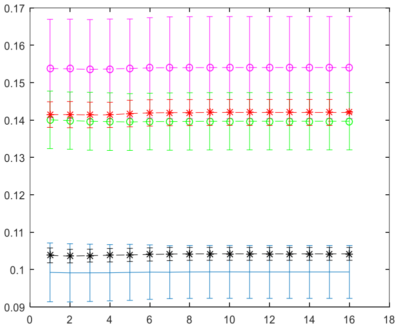

S1 Fig.: MEMD-enhanced MMSE, –MMRQE and MMShE curves for 100ms, 200ms, 400ms, 800ms, 1sec and 10sec snippets. Other conventions are as in Fig. 1
