# Supplementary material for: Discrimination of emotional states from scalp- and intracranial EEG using multiscale Rényi entropy
Source: PLoS One. 2017 Nov 3;12(11):e0186916. doi: 10.1371/journal.pone.0186916 (PMC5669426; doi:10.1371/journal.pone.0186916)
Supplement: S2 Appendix — (PDF) [file pone.0186916.s002.pdf]

S2: MMRQE plotted in terms of individual IMFs

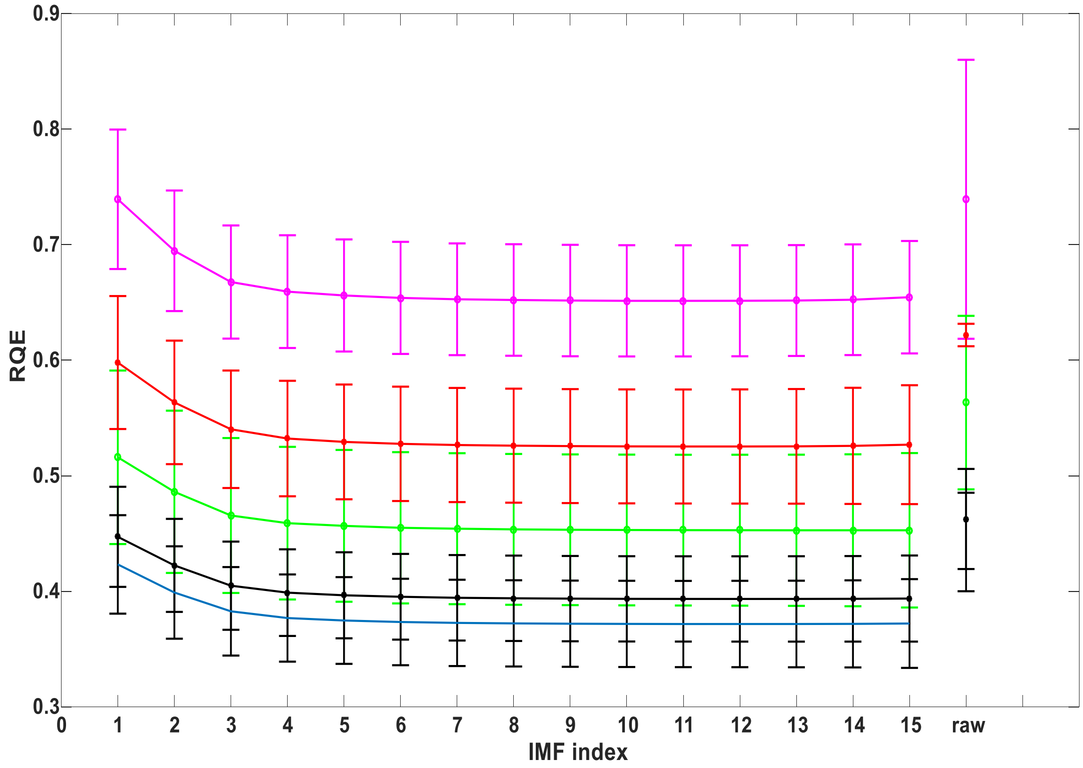

S2 Fig : MEMD-enhanced MRQE plotted as a function of individual IMFs with curves labeled in terms of our 30 participants' self-reports (main experiment) and corresponding uniscale entropy values for the original EEG recordings (labeled as 'raw'). Color convention: purple = anger, red = amusement, green = disgust, black = tenderness, blue = sadness
