# Supplementary material for: Discrimination of emotional states from scalp- and intracranial EEG using multiscale Rényi entropy
Source: PLoS One. 2017 Nov 3;12(11):e0186916. doi: 10.1371/journal.pone.0186916 (PMC5669426; doi:10.1371/journal.pone.0186916)
Supplement: S3 Appendix — (PDF) [file pone.0186916.s003.pdf]

S3: Scalp distribution of original EEG’s alpha coefficient

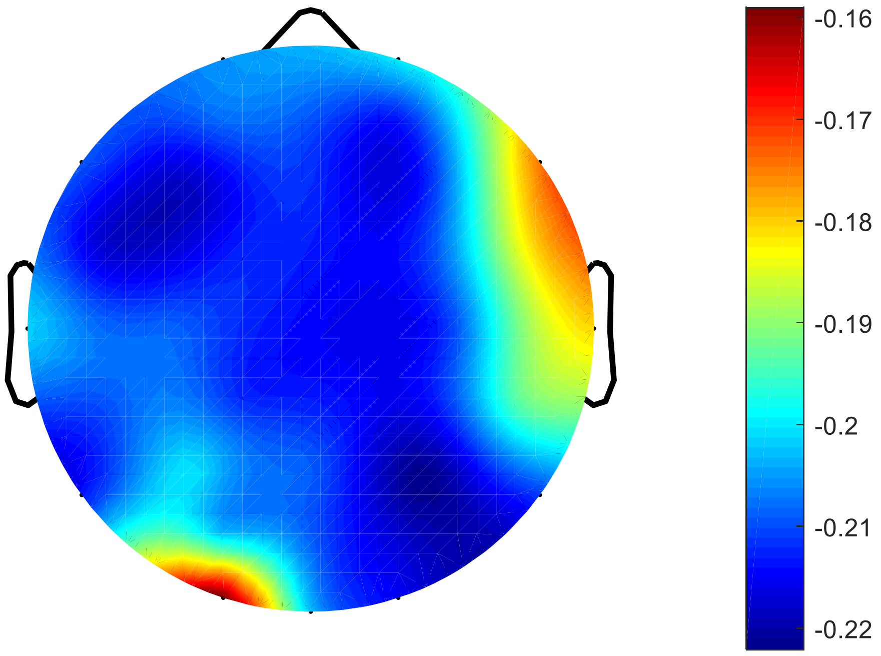

S3 Fig. : Scalp distribution of Krippendorf's alpha coefficient between main group’s univariate, multiscale RQEs based on original (uniscale) EEG recordings
