# Supplementary material for: Discrimination of emotional states from scalp- and intracranial EEG using multiscale Rényi entropy
Source: PLoS One. 2017 Nov 3;12(11):e0186916. doi: 10.1371/journal.pone.0186916 (PMC5669426; doi:10.1371/journal.pone.0186916)
Supplement: S4 Appendix — (PDF) [file pone.0186916.s004.pdf]

S4 : Correlation matrix of depth electrode

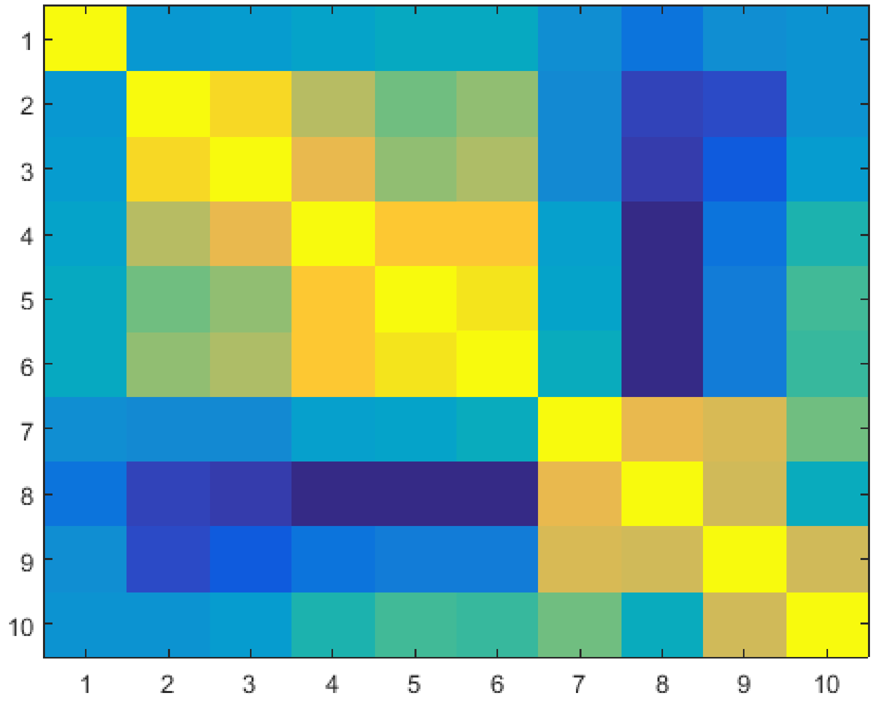

S4 Fig. : Correlation matrix of 10 contact point recordings in response to entire anger video clip
