# Supplementary material for: Discrimination of emotional states from scalp- and intracranial EEG using multiscale Rényi entropy
Source: PLoS One. 2017 Nov 3;12(11):e0186916. doi: 10.1371/journal.pone.0186916 (PMC5669426; doi:10.1371/journal.pone.0186916)
Supplement: S5 Appendix — (PDF) [file pone.0186916.s005.pdf]

S5 : Location of subdural electrode grid

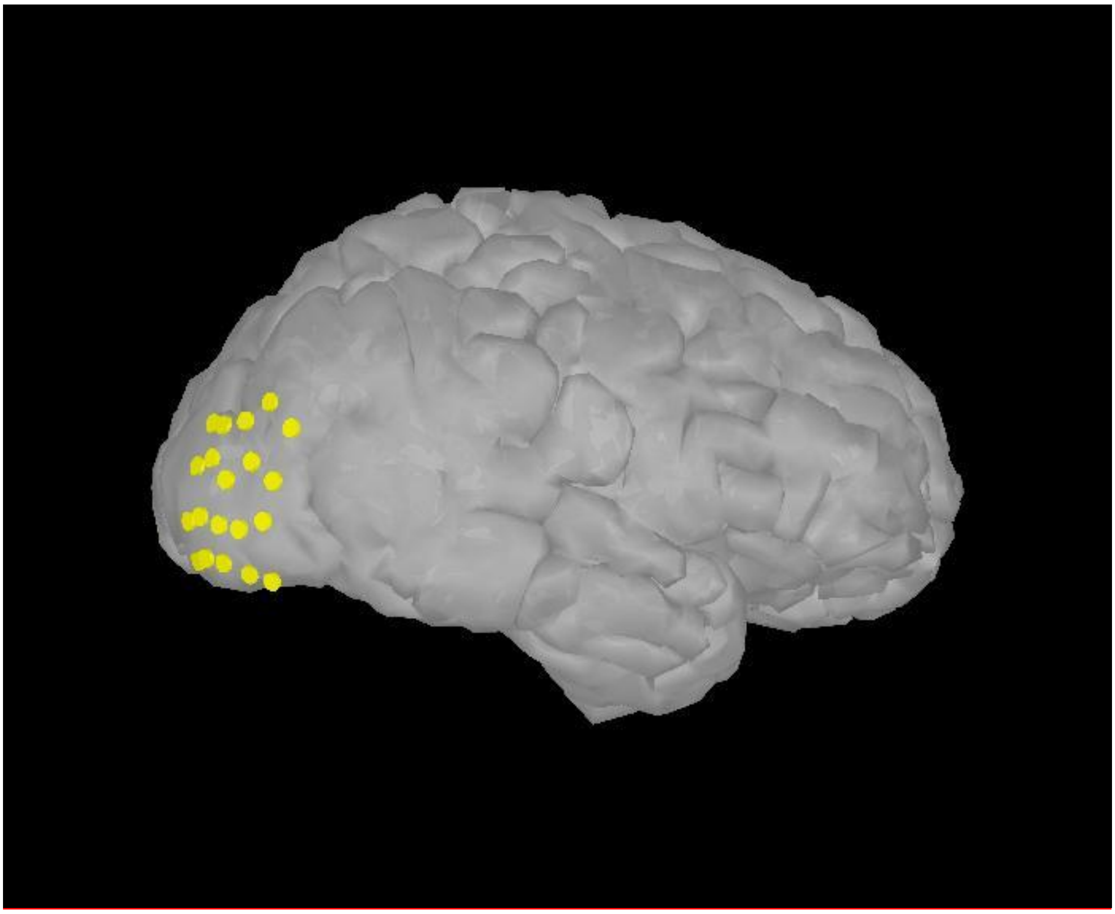

S5 Fig.: 4x5 subdural electrode grid covering right occipital pole
