# Supplementary material for: Discrimination of emotional states from scalp- and intracranial EEG using multiscale Rényi entropy
Source: PLoS One. 2017 Nov 3;12(11):e0186916. doi: 10.1371/journal.pone.0186916 (PMC5669426; doi:10.1371/journal.pone.0186916)
Supplement: S6 Appendix — (PDF) [file pone.0186916.s006.pdf]

S6 MMRQE results for non-French speaking subjects, main experiment

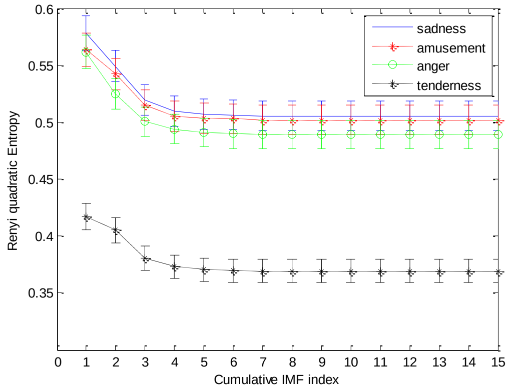

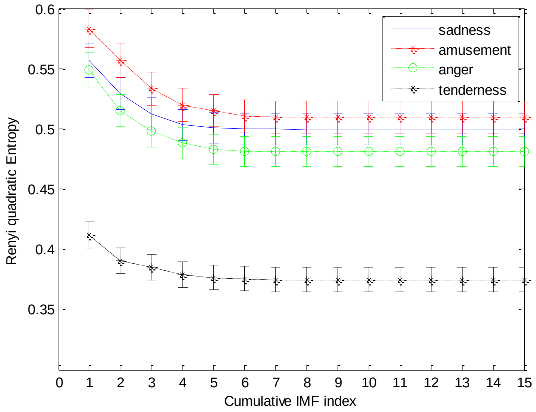

S6 Fig. : MEMD-enhanced MMRQE curves of 2 non-French speaking subjects in response to the video clips used in the main experiment (Table 1). Insert shows the color convention of the curves in terms of the subjects' self-labels. Error bars are standard errors of average MMRQE. Other conventions are as in Fig. 1.
