# Supplementary material for: Discrimination of emotional states from scalp- and intracranial EEG using multiscale Rényi entropy
Source: PLoS One. 2017 Nov 3;12(11):e0186916. doi: 10.1371/journal.pone.0186916 (PMC5669426; doi:10.1371/journal.pone.0186916)
Supplement: S8 Appendix — (PDF) [file pone.0186916.s008.pdf]

S8_Appendix: Phase synchrony between simulated and mid-frontal EEG

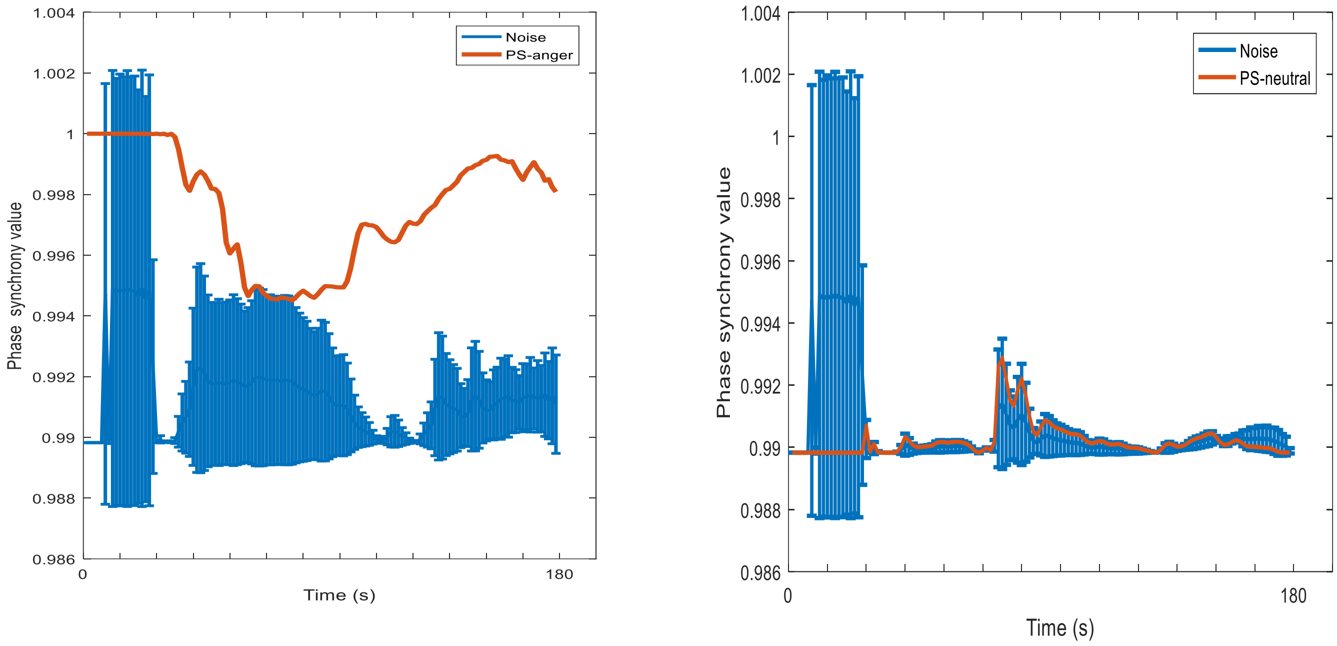

S8 Fig. : Phase synchrony between simulated- (for mixed amygdala dipole orientation case) and recorded mid-frontal EEG. Shown are the results for the emotional ('anger') (red curve, left panel) and neutral video clips (red curve, right panel) and for the corresponding surrogate signal distributions (blue)
